# PPARγ agonists negatively regulate αIIbβ3 integrin outside‐in signaling and platelet function through up‐regulation of protein kinase A activity

**DOI:** 10.1111/jth.13578

**Published:** 2017-02-07

**Authors:** A. J. Unsworth, N. Kriek, A. P. Bye, K. Naran, T. Sage, G. D. Flora, J. M. Gibbins

**Affiliations:** ^1^ Institute for Cardiovascular and Metabolic ResearchSchool of Biological SciencesUniversity of ReadingReadingUK

**Keywords:** blood platelets, platelet activation, platelet glycoprotein GPIIb‐IIIa complex, PPAR gamma, protein kinase A

## Abstract

**Essentials**

- peroxisome proliferator‐activated receptor γ (PPARγ) agonists inhibit platelet function.
- PPARγ agonists negatively regulate outside‐in signaling via integrin αIIbβ3.
- PPARγ agonists disrupt the interaction of Gα13 with integrin β3.
- This is attributed to an upregulation of protein kinase A activity.

**Summary:**

**Background**

Agonists for the peroxisome proliferator‐activated receptor (PPARγ) have been shown to have inhibitory effects on platelet activity following stimulation by GPVI and GPCR agonists.

**Objectives**

Profound effects on thrombus formation led us to suspect a role for PPARγ agonists in the regulation of integrin αIIbβ3 mediated signaling. Both GPVI and GPCR signaling pathways lead to αIIbβ3 activation, and signaling through αIIbβ3 plays a critical role in platelet function and normal hemostasis.

**Methods**

The effects of PPARγ agonists on the regulation of αIIbβ3 outside‐in signaling was determined by monitoring the ability of platelets to adhere and spread on fibrinogen and undergo clot retraction. Effects on signaling components downstream of αIIbβ3 activation were also determined following adhesion to fibrinogen by Western blotting.

**Results**

Treatment of platelets with PPARγ agonists inhibited platelet adhesion and spreading on fibrinogen and diminished clot retraction. A reduction in phosphorylation of several components of αIIbβ3 signaling, including the integrin β3 subunit, Syk, PLCγ2, focal adhesion kinase (FAK) and Akt, was also observed as a result of reduced interaction of the integrin β3 subunit with Gα13. Studies of VASP phosphorylation revealed that this was because of an increase in PKA activity following treatment with PPARγ receptor agonists.

**Conclusions**

This study provides further evidence for antiplatelet actions of PPARγ agonists, identifies a negative regulatory role for PPARγ agonists in the control of integrin αIIbβ3 outside‐in signaling, and provides a molecular basis by which the PPARγ agonists negatively regulate platelet activation and thrombus formation.

## Introduction

Platelets play a vital role in the prevention of blood loss following vascular injury. Through a balance of inhibitory and activating signals, circulating platelets are maintained in a quiescent state in the undamaged circulation and are only activated when signals such as exposed subendothelial matrix proteins at sites of vascular injury outweigh endogenous inhibitory signaling. Platelets adhere rapidly to the site of injury and become activated, characterized by granule secretion, formation of thromboxane A2 and activation of integrin αIIbβ3, which is converted from a low to a high‐affinity conformation, enabling it to bind to fibrinogen and von Willebrand factor (VWF). This initial activation is followed by platelet shape change and aggregation that is supported by fibrinogen binding to the high‐affinity integrin αIIbβ3. Formation of a fibrin coat and clot retraction, which is driven through integrin αIIbβ3‐stimulated outside‐in signaling, then stabilizes the growing thrombus 1.

Inhibitory mechanisms are essential for preventing the formation of unwanted thrombi and for limiting thrombus growth and size. Platelets are inhibited through a number of endogenous mechanisms, including by nitric oxide and prostacyclin released from the healthy endothelium 2 and via signaling receptors on the cell surface such as PECAM‐1 and G6b 3, 4, 5. A recently identified class of intracellular inhibitory receptors is nuclear receptors. Usually involved in the regulation of the transcription of genes, several nuclear receptors have been identified in human platelets and have been shown to play a role in the regulation of platelet activity 2, 3, 6, 7, 8, 9, 10. One such receptor is the peroxisome proliferator‐activated receptor‐γ (PPARγ), a member of the nuclear hormone superfamily 8, 10. In nucleated cells, the PPARγ receptor is known to function in the regulation of many metabolic pathways, including lipid and glucose metabolism and homeostasis 11, 12, 13. Recent studies suggest that the actions of nuclear receptors are not restricted to gene transcription because increasing evidence supports non‐genomic actions of these receptors 14, 15. Studies using platelets support non‐genomic roles for the PPARγ receptor 7, 9, 12, 16, 17. Agonists for PPARγ, such as thiazolidinediones that are in use as a treatment for type 2 diabetes mellitus, have been shown to have inhibitory effects on platelet signaling and activation 6, 7, 8, 18, 19, which could underlie the reported reduction in atherosclerosis, reduced inflammation and cardio‐protective effects in patients treated with PPARγ agonists 12, 13, 16, 20, 21, 22, 23, 24.

We have previously observed the ability of PPARγ agonists to interfere with GPVI‐proximal signaling, but this cannot explain the PPARγ agonist‐dependent inhibition observed following stimulation of platelets with thrombin and other GPCR agonists. Because the integrin αIIbβ3 outside‐in signaling pathway shares several components with the GPVI signaling pathway 25, and is essential for GPCR‐induced platelet activation, we investigated whether the PPARγ receptor is involved in the regulation of integrin αIIbβ3 signaling and the mechanisms by which its agonists negatively regulate platelet function.

The results described here support a role for PPARγ in the negative regulation of integrin αIIbβ3 outside‐in signaling through the up‐regulation of PKA activity and subsequent inhibition of phosphorylation of β3 and downstream components of the integrin αIIbβ3 signaling pathway.

## Materials and methods

### Reagents

Fibrinogen, bovine thrombin, H89, GW9662 and IMBX were purchased from Sigma Aldrich (Poole, UK). 15dPGJ2 was purchased from Enzo Life Sciences and Ciglitazone and SQ22536 from Tocris Bioscience (Bristol, UK). The cAMP ELISA kit was from Enzo Life Sciences (Exeter, UK). Primary anti‐ FAK (focal adhesion kinase) (C20), Syk (N‐19), PLCγ2 (Q20), β3 (C20) and actin (C11) antibodies were purchased from Santa Cruz Biotechnology (Heidelberg, Germany). Phospho‐specific primary antibodies for β3 Y779 and Akt S473 were from Abcam (Cambridge, UK). Anti‐phospho–PKC (protein kinase C) substrate, phospho‐S157 and S239 VASP and phospho‐Ser 19 myosin light chain antibodies were purchased from New England BioLabs (Cell Signalling, Hitchin, UK), and anti‐phospho‐Tyr 4G10 antibody was purchased from Millipore (Watford, UK). Fluorophore conjugated secondary antibodies, Fluo‐4 calcium indicator dye and Alexa‐488 and Alexa‐647 conjugated phalloidin were purchased from Life Technologies (Paisely, UK). All other reagents were from previously described sources 26.

### Human washed platelet preparation

Human blood was obtained from consenting aspirin‐free healthy volunteers, in accordance with the procedures approved by the University of Reading Research Ethics Committee. Blood was collected into 4% (w/v) sodium citrate before mixing with acid citrate dextrose (29.9 mm Na_3_C_6_H_5_O_7_, 113.8 mm glucose, 72.6 mm NaCl and 2.9 mm citric acid [pH 6.4]). Washed platelets were prepared by centrifugation at 100 × *g* for 20 min, followed by centrifugation twice at 1000 × *g* for 10 min in the presence of 1.25 μg mL^−1^ prostacyclin (PGI_2_) as described previously 27. Platelets were resuspended in modified Tyrode's‐HEPES buffer (134 mm NaCl, 0.34 mm Na_2_HPO_4_, 2.9 mm KCl, 12 mm NaHCO_3_, 20 mm N‐2‐hydroxyethylpiperazine‐N‐2‐ethanesulfonic acid, 5 mm glucose and 1 mm MgCl_2_, pH 7.3) and rested for 30 min at 30 °C before use.

### Spreading on fibrinogen

Washed platelets (2 × 10^7^ mL^−1^), pretreated with PPARγ agonists or vehicle control (0.1% v/v dimethylsulfoxide [DMSO]), were exposed to fibrinogen (100 μg mL^−1^) coated coverslips (blocked with 1% bovine serum albumin [BSA]) and incubated for 45 min at 37 °C. Non‐adherent platelets were removed and coverslips washed with phosphate buffered saline (PBS) before fixing using 0.2% (v/v) paraformaldehyde solution. Platelets were permeabilised in 0.1% (v/v) Triton‐X100 prior to staining with Alexa 488 conjugated‐phalloidin for 1 h. Adherent platelets were then imaged with a 100x magnification oil immersion lens on a Nikon A1‐R confocal microscope. Adhesion and spreading data in each experiment were measured by counting the number of platelets and the extent of spreading in five fields of view chosen randomly from each sample.

### Clot retraction assay

Human washed platelets at 5 × 10^8^ mL^−1^ were added to aggregometer tubes in the presence of 2 mg mL^−1^ fibrinogen and 2 mm CaCl_2_ and preincubated with 15dPGJ2 or vehicle control (0.1% v/v DMSO). Clot retraction was initiated by adding an equal volume of 2 U mL^−1^ thrombin and left for 1 h at room temperature before the weight of the clot was measured.

### Adhesion on collagen under flow

Adhesion on collagen in the presence of integrillin (10 μm) was studied *in vitro* using microfluidic flow cells (Vena8, Cellix Ltd, Dublin, Ireland) as described previously 28. DiOC6 loaded human whole blood with or without treatment, in the presence of integrillin (10 μm), was perfused through collagen‐coated (100 μg mL^−1^) Vena8Biochips at a shear rate of 20 dyn cm^−2^. Platelet adhesion was determined by comparing fluorescence intensity in the vehicle and treated samples.

### Immunoblotting and immunoprecipitation

Following adhesion to fibrinogen or stimulation with thrombin (0.1 U mL^−1^) for 5 min under non‐stirring conditions, washed platelets (4 × 10^8^ cells mL^−1^) were lysed in an equal volume of NP40 buffer (300 mm NaCl, 20 mm Tris base, 2 mm EGTA, 2 mm EDTA, 1 mm PMSF, 10 μg mL^−1^ aprotinin, 10 μg mL^−1^ leupeptin, 0.7 μg mL^−1^ pepstatin A, 2 mm sodium orthovanadate, 2% (v/v) NP‐40, pH 7.3) and proteins of interest isolated by immunoprecipitation using 1 μg mL^−1^ of appropriate antibodies. Prior to immunoblotting, which was performed as described previously 7, the lysates of adhered washed platelets were corrected for the level of adhesion by determining the protein concentration of each sample. Proteins were detected using fluorophore‐conjugated secondary antibodies and visualized using a Typhoon FLA 9500 Fluorimager and Image Quant software (GE Healthcare, Chalfont, Buckinghamshire, UK). Band intensities were quantified and levels of the immunoprecipitated protein or loading control were measured and used to normalize the phosphorylation data for protein loading levels.

### Statistical analysis

Statistical analyses were performed on data using GraphPad prism software (GraphPad Software, San Diego, CA, USA). Data were analyzed using Student's *t*‐test and one‐way analysis of variance (anova). Values obtained in several experiments were converted into percentages for comparison of controls with treated samples. *P* ≤ 0.05 was considered statistically significant. Unless stated otherwise, values are expressed as mean ± SEM; *n* values are ≥ 3.

## Results

### PPARγ agonists negatively regulate adhesion and spreading on fibrinogen

**Figure 1:**
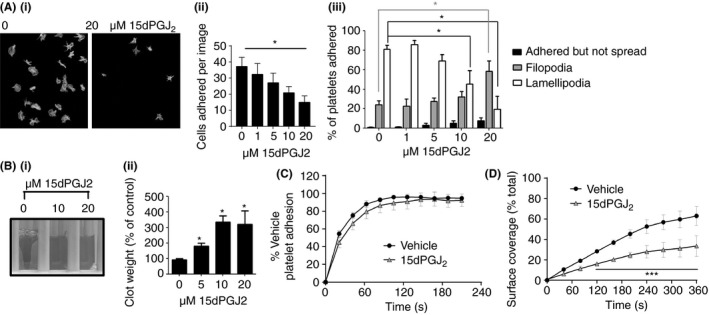
The effect of the peroxisome proliferator‐activated receptor γ (PPARγ) agonists on outside‐in signaling. Human washed platelets pretreated for 10 min with or without increasing concentrations of 15dPGJ2 (1, 5, 10 and 20 μm) or vehicle control were exposed to fibrinogen (100 μg mL^−1^)‐coated coverslips. (A) (i) Representative images of spreading and adhesion after 45 min in vehicle and treated samples. Platelets were stained with phalloidin Alexa‐488 for visualization. Images were taken under oil immersion with magnification × 100. (ii) Adhesion: number of platelets adhered were counted in five randomly selected fields of view for each experiment and the number of cells adhered expressed as a percentage of the vehicle treated control. (iii) Spreading: platelets were classified into three different categories to determine the extent of their spreading (Adhered but not spread. Filopodia: platelets in the process of extending filopodia. Lamellipodia: platelets in the process of extending lamellipodia, including those fully spread). At least 100 platelets of each type were scored. Results expressed as relative frequency, as a percentage of the total number of platelets adhered. (B) Clot retraction. Human washed platelets pretreated for 10 min with or without increasing concentrations of 15dPGJ2 (5, 10 and 20 μm) or vehicle control were added to aggregometer tubes in the presence of 2 mg mL^−1^ fibrinogen and 2 mm CaCl_2_. Clot retraction was initiated by adding 1 U mL^−1^ thrombin and left to proceed for 1 h at room temperature. Clot retraction was determined by weighing the clot. (i) Representative images using red blood cell stained platelet rich plasma (shown). (ii) Data expressed as percentage of vehicle treated control. (C and D) DiOC6 loaded human whole blood was pretreated with vehicle (circles) or 20 μm 15dPGJ2 (triangles) in the presence of integrillin (10 μm) to prevent platelet aggregation for 5 min before (C) perfusion through collagen‐coated (100 μg mL^−1^) Vena8Biochips or (D) perfusion through fibrinogen‐coated (100 μg mL^−1^) Vena8Biochips at a shear rate of 20 dyn cm^−2^. Platelet adhesion and surface area coverage were determined after 5 min by comparing fluorescence intensity in the vehicle and treated samples. Unless stated otherwise, results represent mean + SEM for *n* ≥ 3. *Indicates *P* ≤ 0.05 in comparison with vehicle controls.

Following fibrinogen binding and clustering of integrin αIIbβ3, outside‐in signaling is initiated, leading to platelet shape change and spreading. To determine whether the PPARγ receptor plays a role in the regulation of outside‐in signaling through integrin αIIbβ3, adhesion and spreading on fibrinogen (100 μg mL^−1^) were analyzed in the presence and absence of PPARγ receptor agonists. Additional platelet agonists were not added, to enable the study to focus (primarily) on outside‐in signaling through αIIbβ3. As shown in Fig. 1, treatment of platelets with increasing concentrations of the endogenous PPARγ agonist 15dPGJ2 caused a significant decrease in the ability of the platelets to adhere to and spread on fibrinogen when compared with vehicle‐treated controls. 15dPGJ2 (20 μm) caused ~ 50% inhibition of adhesion and a significant reduction in spreading. A reduction in surface area coverage was observed following treatment with increasing concentrations of 15dPGJ2 (data not shown) and ~50% fewer platelets generated lamellipodia compared with vehicle control (0.1% v/v DMSO). Inhibition of both adhesion and spreading was also observed in the presence of inhibitors of ADP and thromboxane A2 (Figure S1A), which is in agreement with previously published data showing PPARγ agonist‐induced inhibition of aggregation is independent of regulation of the release of secondary mediators 7. Longer time‐courses for adhesion and spreading were also studied, to test whether the defect in spreading was a result of reduced adhesion kinetics. As shown in Figure S1(B), a delay in platelet adhesion kinetics is observed following treatment with 15dPGJ2, where after 90 min the number of adhered platelets treated with 15dPGJ2 was similar to the number of vehicle‐treated platelets adhered at 45 min. Treatment of platelets with two alternative synthetic PPARγ agonists, ciglitazone and rosiglitazone (20 μm), both thiazolidinediones, also caused inhibition of both adhesion and spreading on fibrinogen compared with the untreated control (Figure S2). In contrast to the PPARγ agonists 15dPGJ2 and Ciglitazone, the PPARγ antagonist GW9662 (10 μm) did not alter platelet adhesion or spreading on fibrinogen under static conditions compared with vehicle control (Figure S2C).

### 15dPGJ2 inhibits platelet clot retraction

Outside‐in αIIbβ3 signaling is essential for the process of clot retraction that is required for thrombus stabilization. As our studies of adhesion and spreading indicate a role in integrin αIIbβ3 outside‐in signaling, the effect of 15dPGJ2 (Fig. 1B), ciglitazone and rosiglitazone (Figure S2Aiv, Biv) on clot retraction was explored. Pre‐incubation of platelets with all three PPARγ ligands resulted in an increase in clot weight and therefore an inhibition of clot retraction after 90 minutes, compared with vehicle control (0.1% v/v DMSO). These results support a negative regulatory role for the PPARγ receptor agonists in outside‐in signaling mediated via integrin αIIbβ3.

### PPARγ agonists do not alter adhesion to collagen under flow

It has been previously described that treatment of platelets with PPARγ agonists results in a significant inhibition of thrombus formation on collagen under flow conditions 7. As we have shown that PPARγ agonists inhibit integrin αIIbβ3 outside‐in signaling, we sought to determine whether this previously observed reduction in thrombus formation on collagen could be attributed to impaired αIIbβ3 signaling and thrombus instability rather than to reduced adhesion to collagen. Platelets were treated with integrillin, an antagonist of integrin αIIbβ3, to prevent fibrinogen binding, outside‐in signaling and platelet aggregation, and the ability of platelets to adhere to collagen under arterial flow was determined. As shown in Fig. 1(C), no difference in adhesion to collagen (measured as overall fluorescence) was observed in 15dPGJ2‐treated whole blood compared with vehicle control (0.1% v/v DMSO) in the presence of integrillin, suggesting that the early stages of thrombus formation (adhesion) on collagen are unaffected. In contrast, adhesion to fibrinogen under flow was reduced following treatment with 15dPGJ2 (Fig. 1D), further supporting a role for 15dPGJ2 in the negative regulation of outside‐in signaling *in vitro*. We therefore hypothesize that the previously described inhibition of thrombus formation 7 is a result of a reduction in integrin function and outside‐in signaling.

### PPARγ agonists reduce myosin light chain phosphorylation

**Figure 2:**
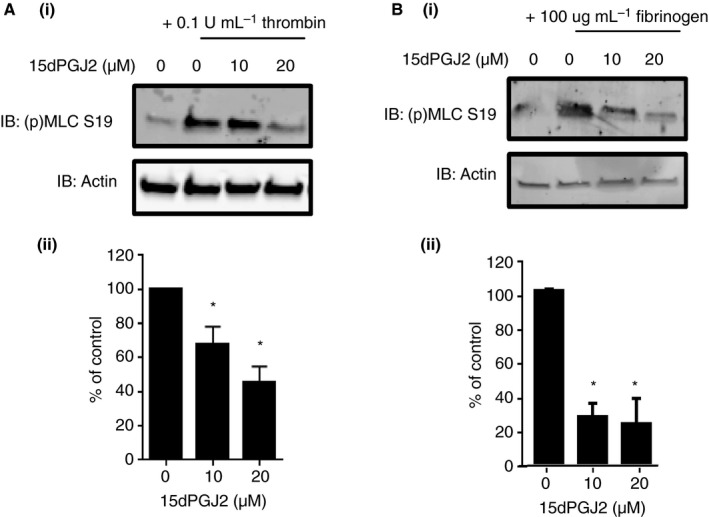
The effect of 15dPGJ2 on phosphorylation of the myosin light chain. Effect of the peroxisome proliferator‐activated receptor γ (PPARγ) agonist 15dPGJ2 (0, 10, 20 μm) on the phosphorylation of the myosin light chain at Ser19 was determined using (A) thrombin‐stimulated (0.1 U mL^−1^) and (B) fibrinogen‐adhered (100 μg mL^−1^) human washed platelets. Platelet lysates were examined by immunoblot analysis using a myosin light chain phospho‐site‐specific antibody for S19. Blots were reprobed for total actin to control for protein loading. (i) Representative blots shown. (ii) Levels of phosphorylation were quantified and expressed as a percentage of vehicle treated controls. Results represent mean + SEM for *n* ≥ 3. *Indicates *P* < 0.05 in comparison with vehicle controls.

Integrin αIIβ3 outside‐in signaling driven platelet shape change and spreading requires cytoskeletal remodeling. A major regulator of this process is myosin IIa. Phosphorylation of the myosin light chain on serine 19 downstream of RhoA enables the interaction of myosin with actin filaments, a process essential for shape change and platelet spreading. We therefore asked whether phosphorylation and activation of the myosin light chain is altered in platelets following treatment with agonists for PPARγ. Pretreatment of human washed platelets with 15dPGJ2 (10, 20 μm) caused a significant reduction in myosin light chain phosphorylation compared with vehicle control (0.1% v/v DMSO) in both thrombin (0.1 U mL^−1^)‐stimulated and fibrinogen (100 μg mL^−1^)‐adhered platelets (Fig. 2). Similar results in thrombin‐stimulated platelets were also achieved following treatment with ciglitazone (20 μm) (Figure S3A). This suggests that PPARγ agonists may act to negatively regulate the components of the integrin αIIbβ3 signaling pathway.

### PPARγ agonist 15dPGJ2 inhibits phosphorylation of integrin β3 and downstream signaling

**Figure 3:**
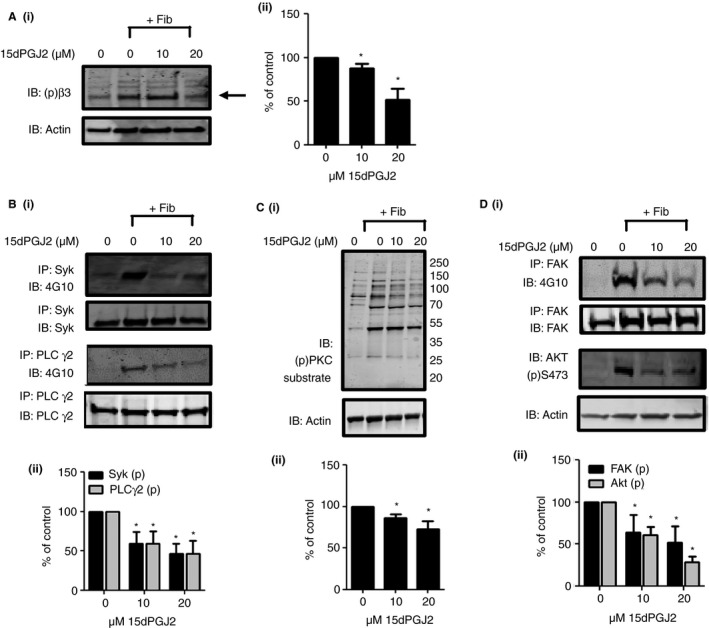
The effect of the peroxisome proliferator‐activated receptor γ (PPARγ) agonist 15dPGJ2 on phosphorylation of integrin β3 signaling components. 15dPGJ2‐treated (0, 10 and 20 μm) human washed platelet lysates were tested for phosphorylation of (A) the integrin β3 tail at Y773, (B) Syk and PLCγ2 phosphorylation, (C) protein kinase C (PKC) substrate phosphorylation and (D) focal adhesion kinase (FAK) and Akt phosphorylation. 15dPGJ2 or vehicle treated platelets were adhered to fibrinogen, lysed in SDS PAGE Laemmli sample buffer, separated on SDS PAGE gels and transferred to poly(vinylidene difluoride) (PVDF) membranes before immunoblotting with a β3 phospho‐site‐specific antibody for Y773, a phospho‐site‐specific antibody for the PKC substrate recognition sequence and an Akt phospho‐site‐specific antibody for S473. Syk, PLCγ2 and FAK were immunoprecipitated from lysates prior to addition of laemmli sample buffer and phosphorylation detected using 4G10 antibody. Blots were reprobed for total β3, Syk, PLCγ2 or actin to confirm equal loading. (i) Representative blots shown. (ii) Levels of phosphorylation were quantified and expressed as a percentage of vehicle treated controls. Results represent mean + SEM for *n* ≥ 3. *Indicates *P* < 0.05 in comparison with vehicle controls.

Phosphorylation of integrin β3 at Y773 (Y747 in mice) is essential for outside‐in signaling by αIIbβ3 29, 30, 31, 32 because phosphorylation at this site is required for the dissociation of talin and association of Gα13, which enables a ‘switch’ from inside‐out to outside‐in signaling 33, 34. The effect of 15dPGJ2 and ciglitazone on phosphorylation of β3 at Y773 was determined in fibrinogen (100 μg mL^−1^)‐adhered platelets. The number of adhered platelets was normalized to ensure any alterations in phosphorylation were not a result of altered adhesion. Adhesion to fibrinogen was associated with an increase in phosphorylation of β3 and, as shown in Fig. 3(A) and Figure S3(B), treatment with either 20 μm 15dPGJ2 or ciglitazone caused a reduction of ~50% and ~30%, respectively, in β3 Y773 phosphorylation in comparison with vehicle control‐treated platelets.

This reduction in β3 phosphorylation may alter the ability of αIIbβ3 to bind to fibrinogen and propagate outside‐in signaling, as previously published data show that PPARγ agonists reduce fibrinogen binding in agonist‐stimulated platelets 7 but may also prevent outside‐in signal transduction following fibrinogen binding to the integrin. In support of the latter a significant reduction (~50%) in both Syk and PLCγ2 phosphorylation (Fig. 3B) was observed in fibrinogen‐adhered washed platelet lysates treated with the PPARγ agonist. Protein kinase C (PKC) is a key mediator of downstream signaling events and its activity is regulated by intracellular calcium levels and production of diacylglycerol. Consistent with the inhibition of PLCγ2, analysis of levels of PKC substrate phosphorylation, a marker of PKC activity, showed a reduction in phosphorylation of approximately 25% in fibrinogen‐adhered platelets that had been pretreated with 15dPGJ2 compared with vehicle controls (Fig. 3C). A similar reduction in the level of PKC substrate phosphorylation was observed following treatment with ciglitazone (data not shown), supporting that these ligands act through PPARγ to negatively regulate integrin αIIbβ3 outside‐in signaling.

### PPARγ agonist‐treated platelets show reduced focal adhesion kinase phosphorylation

A key step in the second wave of cytoskeletal rearrangements following the release of ADP and secondary mediators, is the phosphorylation and activation of focal adhesion kinase (FAK) and PI3‐kinases, which are essential for platelet spreading 35, 36, 37, 38, 39. Treatment of platelets with the PPARγ agonist 15dPGJ2 caused a 50% reduction in phosphorylation of FAK in comparison with controls (Fig. 3D), which is consistent with the ability of the PPARγ agonists to inhibit β3 phosphorylation and platelet shape change and spreading. The effect of 15dPGJ2 on Akt phosphorylation, a key downstream effector of PI3K, was also determined. As shown in Fig. 3(D), fibrinogen‐adhered human washed platelets pretreated with 15dPGJ2 displayed decreased levels of Akt Ser473 phosphorylation, with ~70% inhibition following treatment with 20 μm 15dPGJ2 in comparison with vehicle‐treated controls. These observations further support a negative role for the PPARγ agonists in the regulation of αIIbβ3 outside‐in signaling.

### PPARγ agonists up‐regulate PKA but not PKG activity

Negative regulators of platelet activation can function either by reducing positive signaling or by increasing inhibitory signaling. The cyclic nucleotides cAMP and cGMP, through activation of adenylyl cyclase and cyclic AMP‐dependent protein kinase (PKA) and soluble guanylyl cyclase and cyclic GMP‐dependent protein kinase (PKG), respectively, have both been shown to inhibit adhesion and spreading on fibrinogen and platelet cytoskeletal rearrangements 40, 41. Agonists for the other PPAR receptors, PPARα and PPARβ/δ, have also been shown to inhibit platelets through increasing cAMP levels 17, 42. To determine whether PPARγ agonists achieve inhibition of αIIbβ3 through the up‐regulation of cyclic nucleotide signaling, the effect of PPARγ agonists 15dPGJ2 and ciglitazone on cGMP and cAMP signaling was determined.

**Figure 4:**
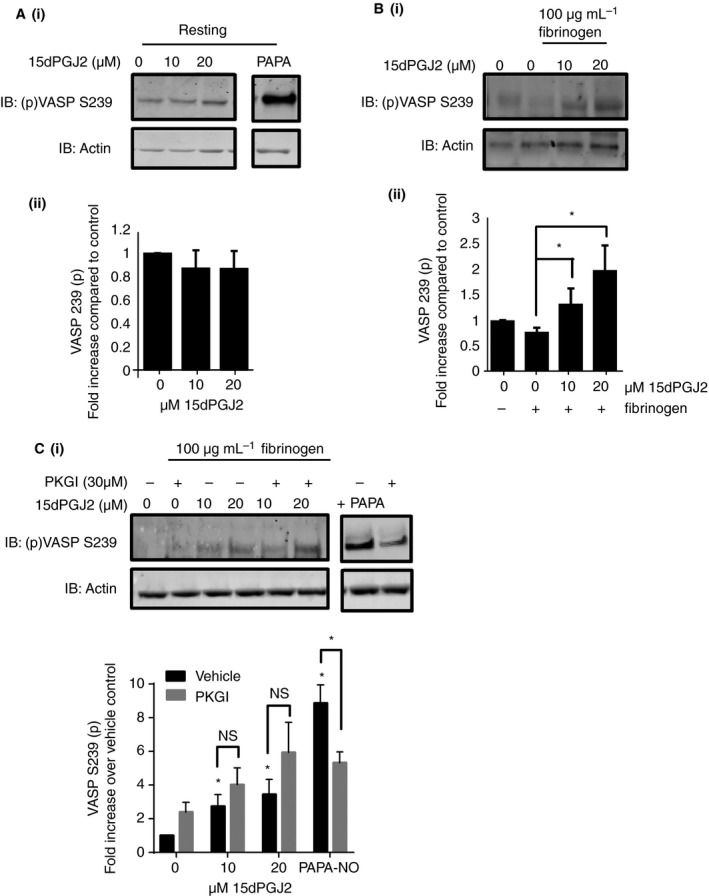
15dPGJ2 does not affect cyclic GMP‐dependent protein kinase (PKG) activity. (A) Resting and (B) fibrinogen‐adhered human washed platelets were treated with increasing concentrations of 15dPGJ2 (10 and 20 μm) or vehicle for 10 min or prior to 45 min of adhesion to fibrinogen (C) in the presence and absence of an inhibitor of PKG, Rp‐8‐Br‐PET‐cGMPS (PKGI) (30 μm), and lysed in laemmli sample buffer. NO‐donor PAPA‐nonoate treated resting platelets were included as a positive control for PKG activation. Platelet lysates were analyzed by immunoblotting for VASP S239 phosphorylation, which is the PKG selective phosphorylation site on VASP. Actin was used to control for protein loading. (i) Representative blots are shown and (ii) levels of phosphorylation were quantified and expressed as fold increase compared with vehicle control. Results represent mean + SEM for *n* ≥ 3. *Indicates *P* < 0.05 in comparison with vehicle controls.

Resting human washed platelets were treated with 15dPGJ2 (10, 20 μm) for 10 min prior to lysis (resting samples) or adhesion to fibrinogen (100 μg mL^−1^). The samples were then analyzed for VASP phosphorylation at Ser239, which is the PKG selective phosphorylation site. Interestingly, although no alteration of VASP S239 phosphorylation was observed in resting platelets (Fig. 4A) an increase in VASP S239 was observed in fibrinogen‐adhered (100 μg mL^−1^) platelets (Fig. 4B) following treatment with 15dPGJ2. This suggested that in fibrinogen‐activated platelets PKG activity is increased following treatment with 15dPGJ2. This increase in VASP S239 phosphorylation in fibrinogen‐adhered platelets was not prevented following treatment with the PKG inhibitor (Rp‐8‐Br‐PET‐cGMPS) (30 μm) at a concentration that was capable of reversing NO donor PAPA‐Nonoate (100 μm) mediated increases in VASP S239, thereby suggesting the increase in VASP S239 phosphorylation is independent of PKG activity (Fig. 4C).

**Figure 5:**
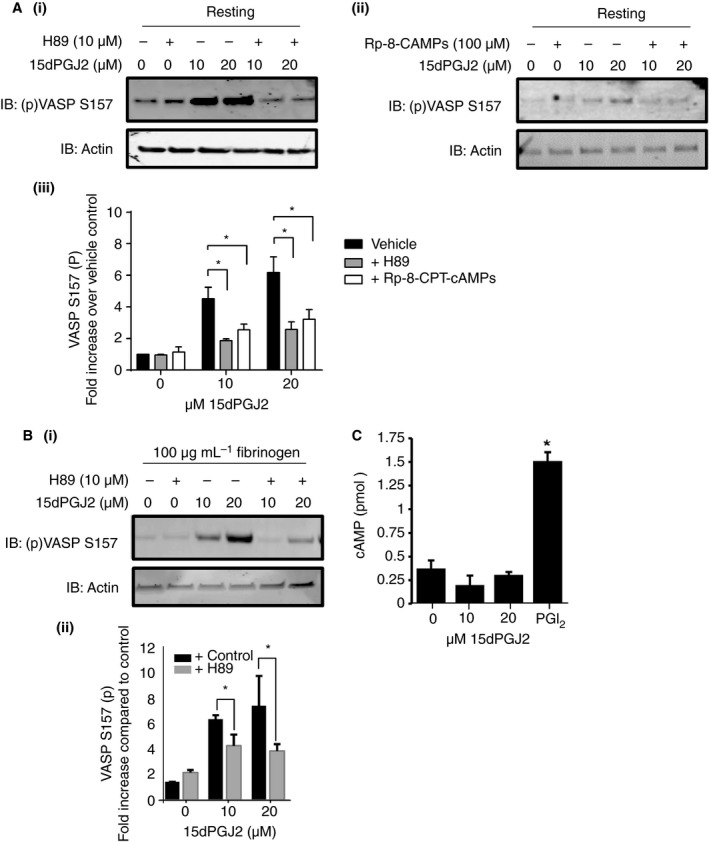
The peroxisome proliferator‐activated receptor γ (PPARγ) agonists alter cyclic AMP‐dependent protein kinase (PKA) activity in platelets. (A) Resting and (B) fibrinogen‐adhered human washed platelets were treated with 15dPGJ2 (10 and 20 μm) in the presence or absence of PKA inhibitor H89 (10 μm) or Rp‐8‐cAMPs (100 μm) for 10 minutes or prior to adhesion to fibrinogen for 45 min and then lysed in laemmli sample buffer. PGI2‐treated resting platelets were included as a positive control for PKA activation. Platelet lysates were analyzed by immunoblotting for VASP S157 phosphorylation, which is the PKA phosphorylation site on VASP. Actin was used to control for protein loading. (A) (i and ii) Representative blots are shown and (iii) levels of phosphorylation were quantified and expressed as fold increase compared with vehicle control. (B) (i) Representative blots are shown and (ii) levels of phosphorylation were quantified and expressed as fold increase compared with vehicle control. (C) Levels of cAMP were determined in 15dPGJ2‐treated (10 and 20 μm) resting washed platelets using a cAMP ELISA assay kit (Enzo) as per the manufacturer's instructions. Results represent mean + SEM for *n* ≥ 3. *Indicates *P* < 0.05 in comparison with vehicle controls.

It has been previously described that PKA can be responsible for phosphorylation of VASP at S239 43. The PKA signaling cascade has also been shown to negatively regulate platelet shape change and spreading through regulation of RhoA 40. The ability of PPARγ agonists to alter PKA activity was therefore determined. As shown in Fig. 5(A, B), treatment with 15dPGJ2 caused a significant increase (8–10‐fold following treatment with 20 μm) in VASP S157 phosphorylation in both resting and fibrinogen‐adhered platelets compared with vehicle alone. This increase in VASP S157 phosphorylation was also observed in ciglitazone‐treated platelets (Figure S3C) and correlated with concentrations of 15dPGJ2 that caused a reduction in MLC S19, β3 Y773 phosphorylation and other integrin αIIbβ3 outside‐signaling components (Figs 2 and 3).

### PPARγ agonists up‐regulate PKA activity

Pretreatment with PKA inhibitors H89 (10 μm) or Rp‐8‐CPT‐cAMPs (100 μm) reduced both 15dPGJ2 and ciglitazone‐mediated increases in VASP S157 phosphorylation in resting and fibrinogen‐adhered platelets (Fig. 5A,B and Figure S3C), supporting our hypothesis that the increase in VASP S157 phosphorylation following treatment with either PPARγ agonist is a result of an increase in PKA activity (Fig. 5A,B).

In addition to PKA, VASP has also been shown to be phosphorylated in platelets by the classical PKC isoforms following stimulation by both GPVI agonists and thrombin 44, 45, 46. PKC activity following treatment of platelets with 15dPGJ2, however, appeared to be unaffected because this was not associated with an increase in PKC substrate phosphorylation (Figure S4A) and treatment with a pan‐PKC inhibitor GF109203X (10 μm) did not prevent the 15dPGJ2 and ciglitazone‐induced increases in VASP S157 phosphorylation. (Figure S4B and C). VASP can also be phosphorylated at S157 by AKT, which is activated downstream of PI3‐kinase, although PPARγ agonist‐induced increases in VASP S157 phosphorylation were not prevented following treatment by either the PI3‐kinase inhibitor LY29400 (100 μm) or AKT inhibitor AKT inhibitor IV (5 μm) (Figure S5), which provides further support that the effect of PPARγ agonists is a result of activation of PKA.

Activation of PKA is widely known to occur following stimulation of Gs coupled receptors, including the prostaglandin receptors: the IP, DP and EP receptors 2. Although more widely known as an endogenous ligand of PPARγ, 15dPGJ2 has also been identified as a possible ligand for the DP1 and DP2 receptors. To rule out that the observed effects were a result of PPARγ ligands activating the prostaglandin receptors, VASP S157 phosphorylation was measured in 15dPGJ2 (10, 20 μm) and ciglitazone (10, 20 μm)‐treated resting platelets in the absence and presence of a DP/EP receptor antagonist AH6809 (10 μm) 47 and IP receptor antagonist Ro1138452 (10 μm) 48, 49. As shown in Figure S6, at concentrations of AH6809 and Ro1138452 that are capable of reversing PGD2 and PGI2‐mediated inhibition of platelet function 47, 49, no reversal of PPARγ ligand‐induced VASP S157 phosphorylation was observed, suggesting that neither 15dPGJ2 nor ciglitazone activates PKA via activation of the prostaglandin receptors (Figure S6).

**Figure 6:**
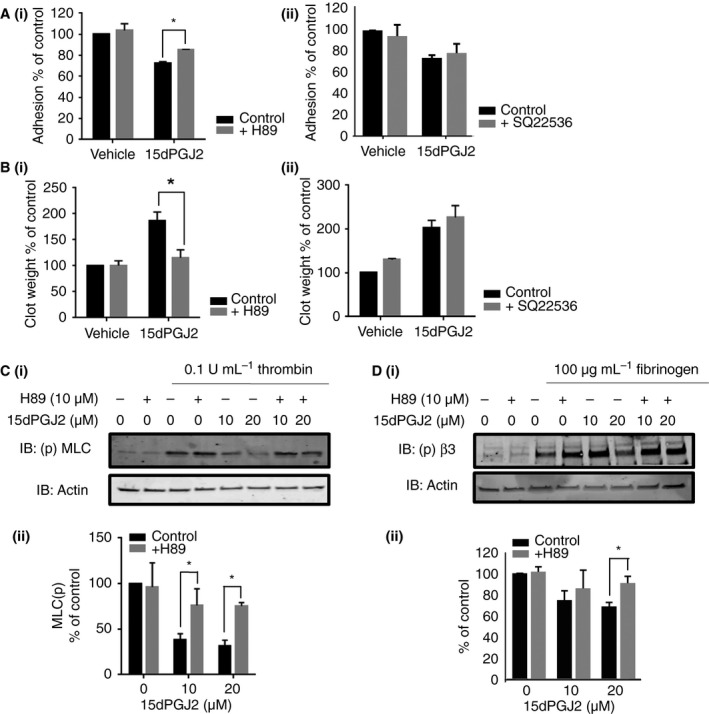
Inhibition of cyclic AMP‐dependent protein kinase (PKA) activity rescues 15dPGJ2‐induced inhibition of outside‐in signaling. Human washed platelets were pretreated for 10 min with 15dPGJ2 (10 and/or 20 μm) or vehicle control in the absence or presence of (i) H89 (10 μm) or (ii) adenylyl cyclase inhibitor SQ22356 (100 μm) before (A) exposure to fibrinogen (100 μg mL^−1^)‐coated coverslips. The number of adhered platelets was expressed as a percentage of the vehicle treated control. (B) Clot retraction assays were performed as described previously. Clot retraction was determined by weighing the clot. (C) Stimulation with 0.1 U mL^−1^ thrombin for 5 min before lysis in laemmli sample buffer and (D) exposure to fibrinogen‐coated coverslips (100 μg mL^−1^) for 45 min. Platelet lysates were analyzed by immunoblotting for (C) myosin light chain phosphorylation using a site‐specific antibody for S19 and (D) β3 phosphorylation using a phospho‐site‐specific antibody for Y773. Actin was used to control for equal loading. (i) Representative blots shown. (ii) Levels of phosphorylation were quantified and expressed as a percentage of vehicle treated control. Results represent mean + SEM for *n* ≥ 3. *Indicates *P* < 0.05 in comparison with vehicle controls.

Interestingly, and in further support of lack of involvement of Gs coupled signaling, no apparent alteration in cAMP levels was observed in resting platelets treated with increasing concentrations of 15dPGJ2 (Fig. 5C), suggesting that the PPARγ agonists primarily regulate platelet activity through the up‐regulation of PKA activity rather than through the alteration of cAMP levels. Furthermore, treatment of platelets with the PKA inhibitor H89 but not the adenylyl cyclase inhibitor SQ22356, reversed 15dPGJ2‐induced inhibition of adhesion to fibrinogen and clot retraction (Fig. 6A and B). Treatment with H89 also reversed 15dPGJ2‐mediated inhibition of both MLC and β3 phosphorylation in thrombin‐ stimulated and fibrinogen‐adhered platelets (Fig. 6C and D).

### PPARγ ligands inhibit integrin β3 interaction with Gα13

**Figure 7:**
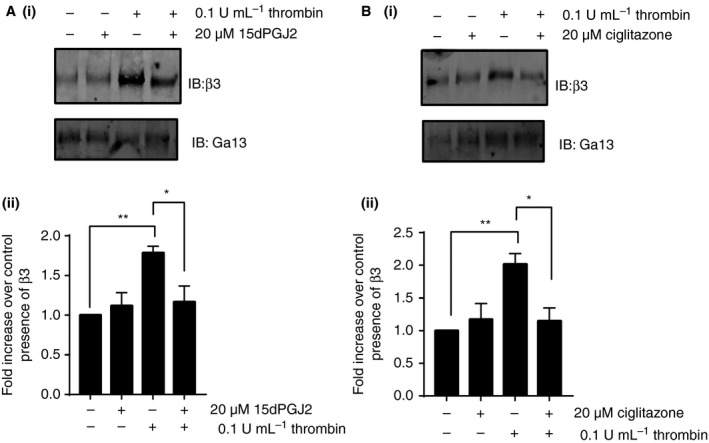
The peroxisome proliferator‐activated receptor γ (PPARγ) ligands inhibit integrin β3 interaction with Gα13. Human washed platelets were pretreated for 10 min with (A) 15dPGJ2, (B) ciglitazone (20 μm) or vehicle control before stimulation with 0.1 U mL^−1^ thrombin for 3 min and reactions stopped by lysis in NP40 lysis buffer. Gα13 was immunoprecipitated from these lysates prior to addition of laemmli sample buffer and the level of interaction with β3 determined by blotting for total β3 and Gα13 in these pull‐down samples. (i) Representative blots shown. (ii) Levels of β3 in each sample were quantified and expressed as a fold change over resting control. Results were normalized to the level of Gα13 present. Results represent mean + SEM for *n* ≥ 3. *Indicates *P* < 0.05 in comparison with vehicle controls.

In addition to RhoA, which is located further downstream in integrin signaling, Gα13 is hypothesized to be phosphorylated and regulated by PKA at a site that could result in conformational changes potentially altering its interaction with its binding partners, including β3 2, 50, 51. Upon binding of the integrin to fibrinogen, Gα13 is thought to bind to the cytoplasmic domain of β3 and is considered to be the directional ‘switch’ that initiates outside‐in signaling 32, 33. To ascertain whether PPARγ ligands disrupt this interaction, the interaction of β3 with Gα13 in thrombin‐stimulated platelets in the presence and absence of PPARγ ligands was determined. β3 was co‐immunoprecipitated with Gα13 using an antibody raised against Gα13. As expected, an increase in the amount of co‐immunoprecipitated β3 was observed following stimulation with thrombin (0.1 U mL^−1^) compared with resting platelets and this interaction was significantly reduced following treatment with either 15dPGJ2 or ciglitazone (Fig. 7).

## Discussion

Outside‐in signaling following fibrinogen binding to integrin αIIbβ3 plays a critical role in platelet function and normal hemostasis and is a necessary secondary activation pathway downstream of several platelet agonists, including both GPVI and GPCR agonists 52.

PPARγ agonists have been shown to have inhibitory effects on both GPVI and thrombin‐evoked inside‐out signaling and platelet activation 6, 7, 8 and it is thought that the PPARγ agonists inhibit GPVI‐induced platelet activation by altering the interaction of PPARγ with, and then phosphorylation of, GPVI signaling proteins, including Syk, LAT and PLCγ2, proteins that are shared by integrin αIIbβ3 outside‐in signaling. We therefore investigated whether PPARγ agonists are involved in the regulation of outside‐in signaling mediated through the platelet integrin αIIbβ3 and hence play a wider role in the regulation of platelet activation.

Binding of integrin αIIbβ3 to and adhesion of platelets on fibrinogen initiates ‘outside‐in’ signaling that leads to cytoskeletal changes involved in the generation and extension of filopodia and lamellipodia and platelet spreading 52. Agonists for PPARγ, including 15dPGJ2, a natural PPARγ agonist, and a synthetic agonist, ciglitazone, caused an inhibition of both platelet adhesion and spreading on fibrinogen in comparison to vehicle‐treated platelets. Clot retraction, another functional process that requires αIIbβ3 outside‐in signaling 53, was also inhibited, providing further evidence that PPARγ agonists regulate processes involved in αIIbβ3 outside‐in signaling. Studies exploring the effect of 15dPGJ2 on platelet adhesion to collagen or fibrinogen under flow established that although treatment with 15dPGJ2 reduced platelet adhesion to fibrinogen, 15dPGJ2 does not alter platelet adhesion to collagen, suggesting that earlier observations of PPARγ agonist‐induced inhibition of thrombus formation 7 could be attributed to reduced αIIbβ3 outside‐in signaling during the later stages of thrombus formation rather than earlier adhesion events.

Upon binding of the integrin to fibrinogen, the G protein subunit Gα13 is thought to bind to the cytoplasmic domain of β3, a process that is enhanced by GPCR signaling 32, 33 and is considered to be the directional ‘switch’ that initiates outside‐in signaling through integrin αIIbβ3. This recruitment of Gα13 to β3 leads to the activation of the Src family kinases (SFKs), including c‐Src, which results in the SFK‐dependent phosphorylation of two tyrosine residues in the β3 tail, Y747 (Y773 in humans) and Y759, which is critical for outside‐in signaling and interaction with other signaling and intracellular molecules. Y747 negatively regulates talin binding and Y759 protects against calpain cleavage 29, 30, 31, 33, 34, 52, 53, 54

In platelets pretreated with the PPARγ agonist 15dPGJ2, a significant reduction in phosphorylation of β3 at Y773 was noted (Fig. 3A), suggesting that the negative regulation of integrin‐αIIbβ3‐dependent processes occurs because of a direct negative regulation of the β3 integrin itself. This negative regulation of β3 signaling by the PPARγ agonists is further supported by attenuation of all downstream signaling (Fig. 3B,C), including phosphorylation and activation of Syk, PLCγ2 and PKC activity, known downstream components of one branch of β3‐dependent c‐Src‐activated signaling 25, 52, 53, 55, 56, and phosphorylation and activation of FAK and PI3K, key mediators in the secondary wave of outside‐in signaling 35, 36, 37, 38 (Fig. 3D). In addition, RhoA signaling, which occurs following activation of c‐Src and is required for cytoskeleton rearrangements, cell spreading and platelet clot retraction 57, was also altered in PPARγ agonist‐treated platelets and myosin light chain phosphorylation (an effector of RhoA activity) was reduced in 15dPGJ2‐treated platelets compared with vehicle controls.

Global inhibition of αIIbβ3 signaling by PPARγ agonists suggests negative regulation occurs upstream in the signaling pathway. However, control of the level of platelet activation is the result of a balance of activatory and inhibitory signaling. Reduction in these activating markers could also be a result of the up‐regulation of endogenous inhibitory signaling pathways. This balance of inhibitory vs. activating signals controls the ability of the platelet to respond to platelet agonists.

A role for PPARγ agonists in pathways known to inhibit αIIbβ3 signaling was also considered. Agonists for other PPARs, including PPARα and PPARβ/δ, have been shown to inhibit platelet activity through increasing cAMP levels and PKA activity 17, 42. The cAMP and PKA signaling cascade has been shown to negatively regulate a number of platelet functions 2, 40, 58, 59, including intracellular calcium mobilization and phosphorylation of Gα13 50, 51, which are important mediators of outside‐in signaling. Platelets treated with either 15dPGJ2 or ciglitazone showed a significant increase in PKA‐mediated VASP S157 phosphorylation that was not associated with activation of the DP, EP or IP prostaglandin receptors or an increase in cAMP levels. In support of this, treatment of platelets with PKA inhibitor H89, but not the adenylyl cyclase inhibitor SQ22536, rescued the PPARγ agonist‐dependent inhibition of platelet adhesion to fibrinogen, clot retraction and integrin αIIbβ3 signaling.

Although the physiological relevance is as yet unclear, studies have shown that the PKA phosphorylation site of Gα13 (T203) is a region that could undergo conformational changes, potentially altering its interaction with its binding partners, including β3 2, 50, 51. We observed that treatment of platelets with PPARγ agonists caused a decrease in Gα13 association with β3, which would prevent c‐Src‐dependent phosphorylation of β3 and dissociation of talin, attenuating signaling downstream of integrin αIIbβ3 (Figure S7). We hypothesize that this inhibition of αIIbβ3 outside‐in signaling could also underlie the observed inhibition of stable thrombus formation previously described *in vitro* and *in vivo* 7, 19.

This study provides further evidence for a role for agonists of nuclear receptors in the negative regulation of platelet activation and supports previous reports that suggest PPARγ agonists are cardio‐protective. PPARγ agonists, thiazolidinediones, are currently used in the treatment of diabetes mellitus type 2. As there is an increased risk of cardiovascular disease associated with diabetes, treatment with PPARγ agonists could complement the effects of other antiplatelet therapies in reducing the risk of thrombosis.

## Addendum

A. J. Unsworth designed the research, performed experiments, analyzed results and wrote the manuscript. N. Kriek and A. P. Bye performed experiments, analyzed results and edited the manuscript. K. Naran performed experiments and analyzed results. T. Sage and G. D. Flora performed experiments. J. M. Gibbins designed the research and wrote the manuscript.

## Disclosure of Conflict of Interests

The authors state that they have no conflict of interest.

## Supporting information


**Fig. S1.** The kinetics of adhesion and spreading on fibrinogen.
**Fig. S2.** PPARγ‐dependent inhibition of outside‐in signaling.
**Fig. S3.** Ciglitazone inhibition of αIIbβ3 outside‐in signaling is associated with up‐regulation of PKA activity.
**Fig. S4.** PPARγ up‐regulation of VASP S157 phosphorylation is not due to activation of PKC.
**Fig. S5.** PPARγ ligand up‐regulation of VASP S157 phosphorylation is not dependent on PI3K or AKT activity.
**Fig. S6.** PPARγ ligand up‐regulation of VASP S157 phosphorylation is not dependent on DP, EP or IP receptor activation.
**Fig. S7.** Negative regulation of αIIbβ3 outside‐in signaling by PPARγ agonists.
