# Supplementary material for: PPARγ agonists negatively regulate αIIbβ3 integrin outside‐in signaling and platelet function through up‐regulation of protein kinase A activity
Source: J Thromb Haemost. 2017 Feb 7;15(2):356–69. doi: 10.1111/jth.13578 (PMC5396324; doi:10.1111/jth.13578)

**PPARγ agonists negatively regulate αIIbβ3 integrin outside-in signalling and platelet function through upregulation of PKA activity.**

**Supplemental figures.**

**Supplementary figure 1. The kinetics of adhesion and spreading on fibrinogen.** A) Human washed platelets pretreated for 10 minutes with increasing concentrations of 15dPGJ2 (20 μM) or vehicle control, in the presence of apyrase (2U/mL) and indomethacin (10 μM), were exposed to fibrinogen (100μg/mL) coated coverslips for 45 minutes. B) Human washed platelets pretreated for 10 minutes with increasing concentrations of 15dPGJ2 (20 μM) or vehicle control, were exposed to fibrinogen (100μg/mL) coated coverslips adhesion and spreading monitored over time (45, 90 and 120 minutes). i) Representative images shown of spreading and adhesion in vehicle and treated samples. Platelets were stained with phalloidin Alexa-488 for visualisation. Images were taken under oil immersion with ×100 lens. ii) Adhesion, the number of platelets adhered were counted per treatment and the average number of cells over 5 images compared between the different time points and conditions. iii) Spreading, platelets were classified into 3 different categories to determine the extent of their spreading (Adhered but not spread, Filopodia: platelets in the process of extending filopodia and Lamellipodia: platelets in the process of extending lamellipodia including those fully 3 spread). Results expressed as a percentage of the total number of platelets adhered. Results represent mean + S.E.M. for n≥3, * indicates p≤0.05 in comparison to vehicle controls.


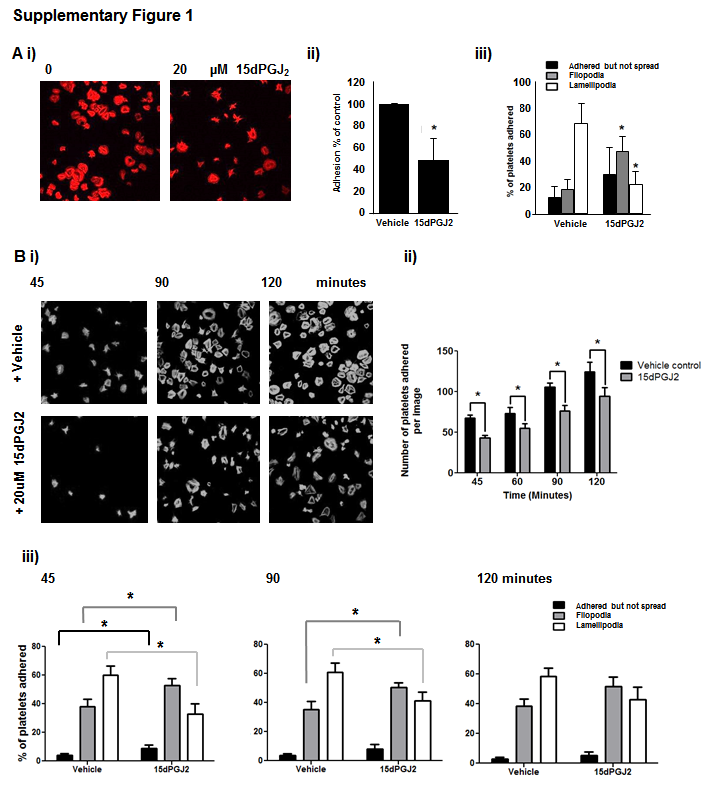


**Supplementary figure 2. PPARγ dependent inhibition of outside in signalling.** Human washed platelets pretreated for 10 minutes with or without increasing concentrations of A) Ciglitazone (1,5,10,20 μM), B) Rosiglitazone (10, 20 µM) C) GW9662 (10 μM) or vehicle control were exposed to fibrinogen (100μg/mL) coated coverslips. A, B i) Representative images shown of spreading and adhesion after 45 min in vehicle and treated samples. Platelets were stained with phalloidin Alexa-488 for visualisation. Images were taken under oil immersion with magnification ×100. A, B ii), C,iii) Adhesion, Number of platelets adhered were counted in 5 randomly selected images in each experiment and the number of cells adhered expressed as a percentage of the vehicle treated control. A, B iii), C,iii) Spreading, platelets were classified into 3 different categories to determine the extent of their spreading (Adhered but not spread, Filopodia: platelets in the process of extending filopodia and Lamellipodia: platelets in the process of extending lamellipodia including those fully spread). Results expressed as relative frequency, as a percentage of the total number of platelets adhered. A,B iv) Clot retraction measured in human washed platelets in aggregometer tubes in the presence of 2 mg/mL fibrinogen and 2 mM CaCl_2_. Clot retraction was initiated by adding 1 U/mL thrombin and left to proceed for 1 hour at room temperature. Clot retraction was determined by weighing the clot. Data expressed as percentage of vehicle treated control. Results represent mean + S.E.M. for n≥3, * indicates p≤0.05 in comparison to vehicle controls.


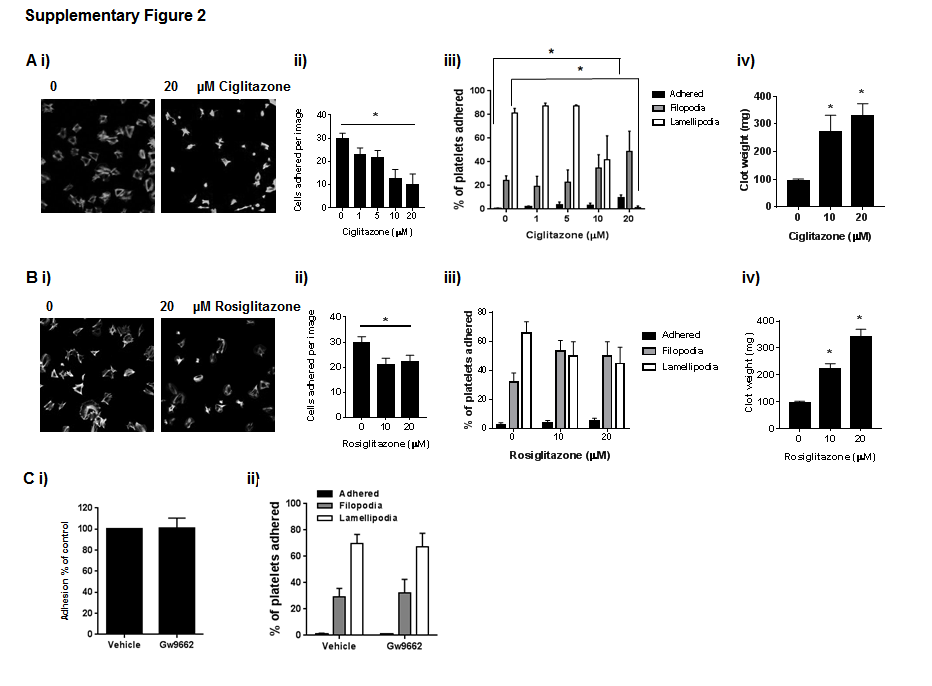


**Supplementary Figure 3. Ciglitazone inhibition of αIIbβ3 outside-in signalling is associated with upregulation of PKA activity.** The effect of the PPARγ agonist Ciglitazone (0, 10, 20 µM) on the phosphorylation of A) the myosin light chain at Ser19 B) integrin β3 at Y747 and C) VASP at S157 was determined using A) thrombin stimulated (0.1 U/mL) and B) fibrinogen adhered (100 µg/mL) human platelets and C) resting unstimulated human washed platelets in the presence or absence of PKA inhibitors i) H89 (10 µM), ii) Rp-8-CPT-cAMPs (100 µM) or vehicle control. Platelet lysates were examined by immunoblot analysis using phospho-site specific antibodies. Blots were reprobed for total actin to control for protein loading. A, B i) C I and ii) representative blots shown, A, B ii) and C iii) Levels of phosphorylation were quantified and expressed as a percentage of vehicle treated controls. Results represent mean + S.E.M. for n≥3, * indicates p<0.05 in comparison to vehicle controls.


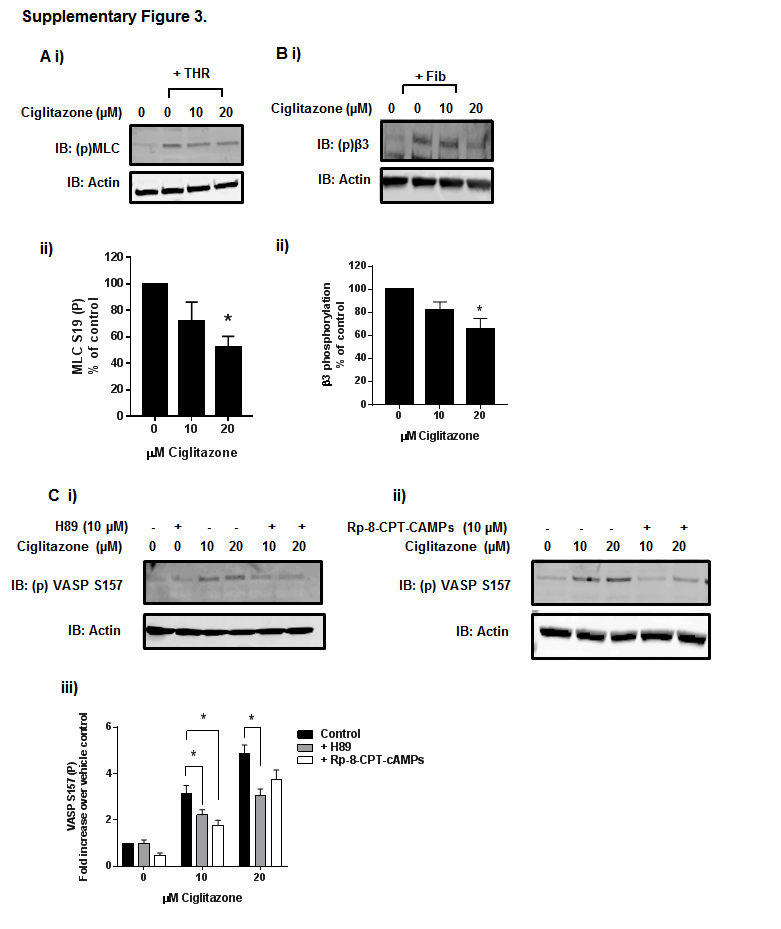


**Supplementary Figure 4. PPARγ upregulation of VASP S157 phosphorylation is not due to activation of PKC.** A) The effect of the PPARγ agonist 15dPGJ2 (0, 10, 20 µM) on basal levels of PKC substrate phosphorylation was determined. Thrombin (0.1 U/mL) was included as a positive control. The effect of the PKC inhibitor GF109030X (10 µM on PPARγ ligand B) 15dPGJ2 or C) Ciglitazone dependent increases in VASP S157 phosphorylation in human resting platelets was determined. Platelet lysates were examined by immunoblot analysis using phospho-site specific antibodies. Blots were reprobed for total actin to control for protein loading. i) representative blots shown, ii) Levels of phosphorylation were quantified and expressed as a percentage of vehicle treated controls. Results represent mean + S.E.M. for n≥3, * indicates p<0.05 in comparison to vehicle controls.


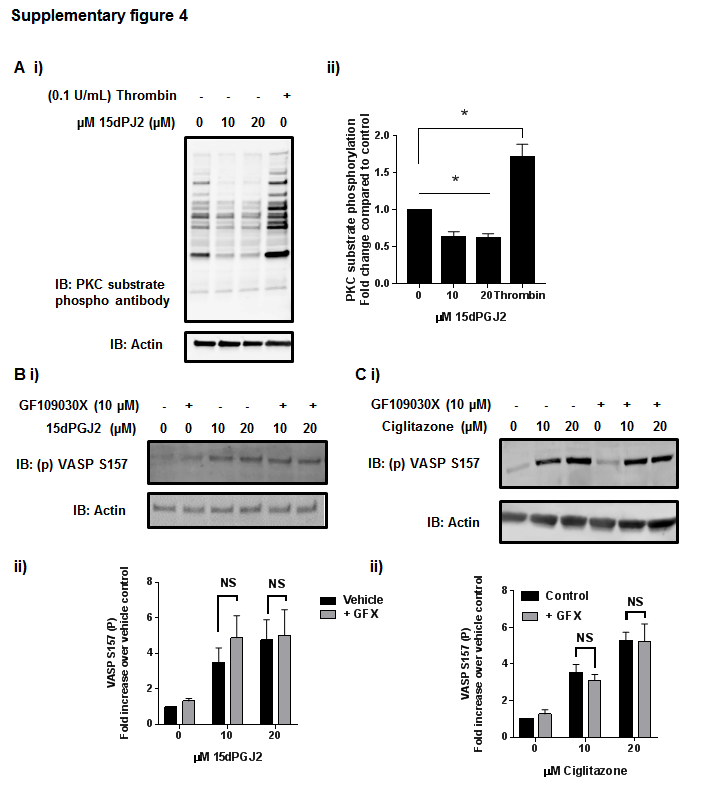


**Supplementary Figure 5. PPARγ ligand upregulation of VASP S157 phosphorylation is not dependent on PI3K or AKT activity.** The effect of the A, B) PI3K inhibitor LY29400 (50 µM) or C,D) AKT inhibitor, AKT inhibitor IV (5 µM) on 15dPGJ2 and Ciglitazone dependent increases in VASP S157 phosphorylation in human resting platelets was determined. Platelet lysates were examined by immunoblot analysis using phospho-site specific antibodies. Blots were reprobed for total actin to control for protein loading. i) representative blots shown, ii) Levels of phosphorylation were quantified and expressed as a percentage of vehicle treated controls. Results represent mean + S.E.M. for n≥3, * indicates p<0.05 in comparison to vehicle controls.


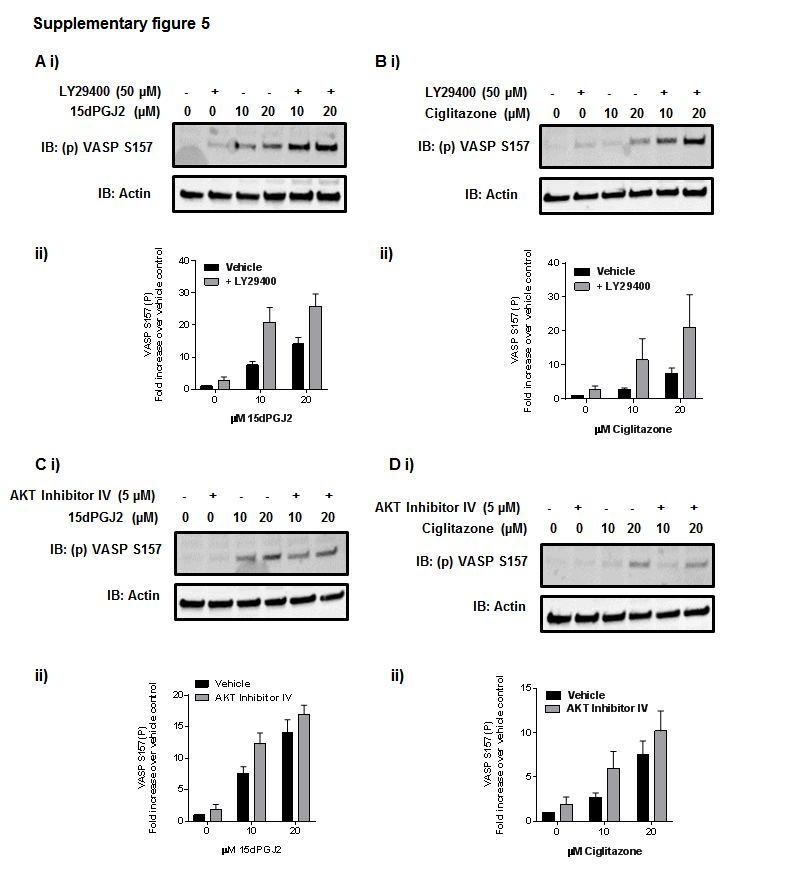


**Supplementary Figure 6. PPARγ ligand upregulation of VASP S157 phosphorylation is not dependent on DP, EP or IP receptor activation.** The effect of the A,B) DP/EP receptor antagonist AH6809 (10 µM) or C,D) IP receptor antagonist, Ro1138452 (10 µM) on 15dPGJ2 and Ciglitazone dependent increases in VASP S157 phosphorylation in human resting platelets was determined. PGD2 and PGI2 the DP and IP receptor agonists were included as positive controls. Platelet lysates were examined by immunoblot analysis using phospho-site specific antibodies. Blots were reprobed for total actin to control for protein loading. i) representative blots shown, ii) Levels of phosphorylation were quantified and expressed as a percentage of vehicle treated controls. Results represent mean + S.E.M. for n≥3, * indicates p<0.05 in comparison to vehicle controls


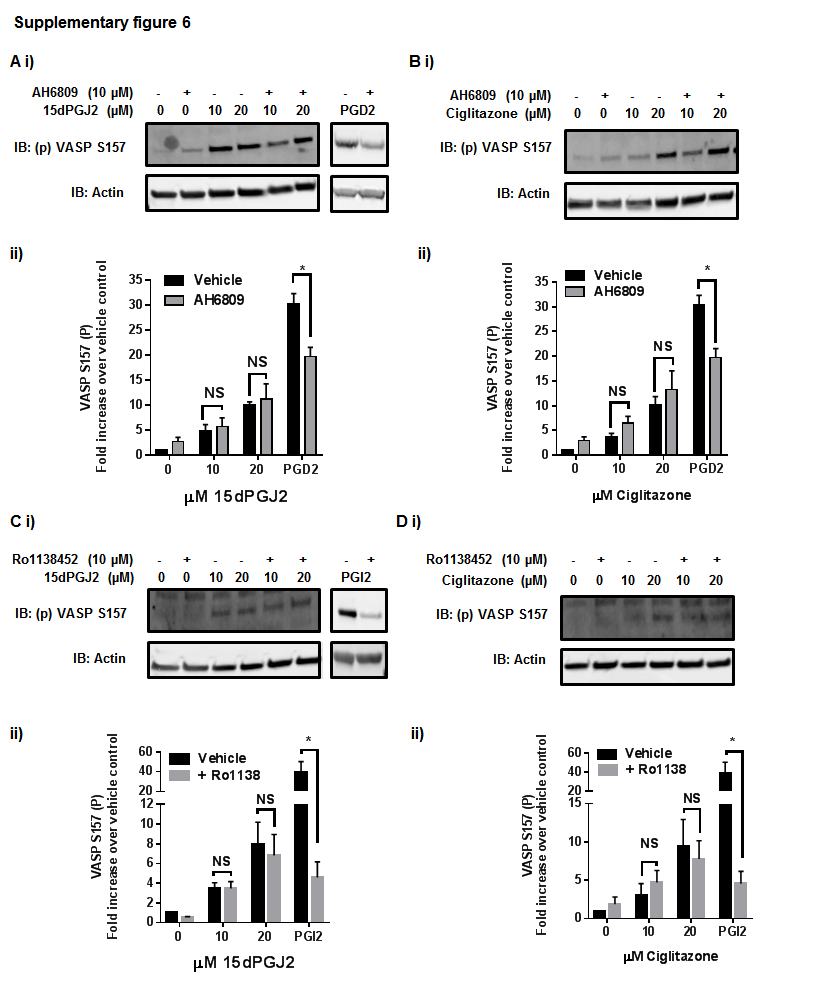


**Supplementary figure 7. Negative regulation of αIIbβ3 outside-in signalling by PPARγ agonists.** A schematic showing the points of negative regulation of integrin mediated outside-in signalling in human washed platelets following treatment with PPARγ agonists. Black arrows indicate the αIIbβ3 outside-in signalling cascade and outcomes, dark grey arrows and crosses indicate the points of regulation by PPARγ agonists and the points of negative regulation. Light grey arrows and crosses indicate previously described PKA points of negative regulation and the dashed line represents an additional hypothesised point of negative regulation of the pathway.


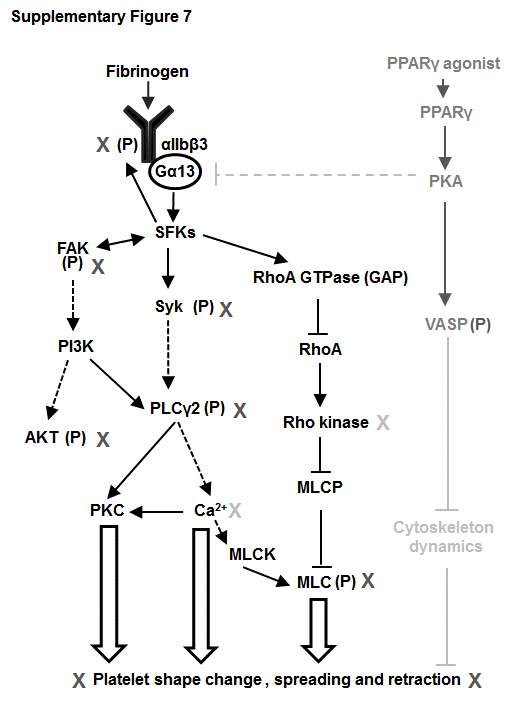

Supplement: Supplementary file 1 — Fig. S1. The kinetics of adhesion and spreading on fibrinogen. Fig. S2. PPARγ‐dependent inhibition of outside‐in signaling. Fig. S3. Ciglitazone inhibition of αIIbβ3 outside‐in signaling is associated with up‐regulation of PKA activity. Fig. S4. PPARγ up‐regulation of VASP S157 phosphorylation is not due to activation of PKC. Fig. S5. PPARγ ligand up‐regulation of VASP S157 phosphorylation is not dependent on PI3K or AKT activity. Fig. S6. PPARγ ligand up‐regulation of VASP S157 phosphorylation is not dependent on DP, EP or IP receptor activation. Fig. S7. Negative regulation of αIIbβ3 outside‐in signaling by PPARγ agonists. [file JTH-15-356-s001.docx]
